# Case of Formation of Pus in the Stomach, and of Wound in the Thumb Caused by the Dissection of the Body, in the Person of W. H. Callow, Esq. Surgeon of the 96th Regiment of Foot

**Published:** 1824-08

**Authors:** 


ART. IV.—*Case of Formation of Pus in the Stomach, and of Wound in the Thumb caused by the Dissection of the Body, in the Person of* W. H. CALLOW, Esq. *Surgeon of the* 96*th Regiment of Foot.*

*Case of Thomas Abell, aged twenty-two, Private Soldier in the* 96*th Regiment.*

THOMAS ABELL reported himself sick on the morning of the 3d of April, complaining of violent pain in epigastrio, with incessant vomiting; pulse 120, small, and hard; tongue loaded, brown, and dry; surface cold; bowels constipated. Eighteen or twenty ounces of purulent matter was discharged from the stomach in my presence. Shortly afterwards, the pain shifted to the vicinity of the umbilicus, where it remained fixed, and the case assumed the unequivocal character of enteritis. Within the next twenty-four hours, depletion to the extent of one hundred ounces was effected, by the lancet and by leeches to the abdomen. Cathartics were repeated, till several evacuations of fecal matter were produced, mixed with which considerable quantities of pus was observed. The warm bath was frequently used, and fomentations constantly applied to the abdomen. On the afternoon of the second day, considerable amendment appeared to have been effected, and some hopes of recovery were entertained, when, in a violent fit of vomiting, the patient suddenly expired.

*Sectio Cadaveris of Thomas Abell.*—Upon view of the body, no tumefaction or inflation of the abdomen was perceptible, nor any external discoloration.

The subject was of full muscular formation, and a considerable layer of fat was found to extend over the external abdominal muscles.

The first division of the peritoneum presented the omentum slightly injected, the small intestines bearing evidence of generally diffused inflammatory action, to a limited degree; no approach to sphacelus, or disorganization of any kind, being to be detected in this part of the canal.

The viscera were found enveloped in pus, which was ultimately discovered to occupy the entire cavity: in quantity, about thirty ounces were extracted; and the quality appeared bland, and of perfect formation.

Proceeding to examine the stomach, pus in large quantity was seen to gush from under it, upon the first interference with its position; a lacerated aperture was briefly discovered in the posterior part, approaching the small curvature, which extended on all sides, upon the most gentle attempts to raise the viscus from its position; a considerable portion of the parietes of which, surrounding the pylorus, and the posterior part of the small curvature, were in a state of complete disorganization.

The contents of the stomach appeared to have been exclusively pus, with one exception; that was, a substance which had assumed the shape of an egg, and was about the dimensions of that of a pullet. It had the appearance of ingesta, possibly cheese; was evidently of a single substance, granulated in texture, without any nucleus, and of a consistence that but barely admitted of removal.

The duodenum was occupied by pus; but the small intestines were void of fæces, or contents of any kind. The canal was not in any part contracted, nor were the intestinal coats indurated or thickened. The evidences of inflammatory action having existed to a considerable extent, were very general.

The liver and other viscera of the abdomen were separately examined, and found of healthy structure; as were equally the contents of the thoracic cavity.

The most extraordinary circumstances attending this case are, that the patient continued to fulfil the duties of hospital-cook till the day of his admission into a sick ward, which was but thirty-four hours prior to his decease; that no antecedent indisposition was complained of, nor the least emaciation produced, while pus, to the extent of at least seven pounds, was forming in one of the most vital organs.

Subsequent inquiries have elicited information, that this patient was habitually disposed to an immoderate use of spirits; that recently he complained to his comrades of great diminution of his accustomed strength, and an inability to retain any food long upon his stomach; but he was never heard to express himself in pain, till the morning he reported himself sick.

It is worthy of remark, that pus, as long as it was contained in a cyst, evidently excited but trifling irritation in the alimentary canal, except when the magnitude of the abscess, and its apparent situation near the pylorus, obstructed the passage into the duodenum. But the moment the rupture of the cyst brought its contents in contact with the mucous tissues, their excitement to a state of active inflammation ensued. There can be but little hesitation in concluding, that the sudden termination of the case was occasioned by the rupture of the attenuated coats of the stomach.

*Case of W. C. Callow, Esq. Surgeon of the 96th Regiment.*

The morbid examination of Thomas Abell occupied a considerable interval, from a desire to place beyond a doubt the situation the abscess had occupied; the parietes of the stomach being generally in such a state of disorganization, that no distinct cyst was apparent: the liver, therefore, and successively each of the abdominal and thoracic viscera, were separately examined.

During a period of more than two hours, the operator's hands were necessarily immersed in the purulent matter, with which every part of the abdominal cavity was deluged. A small puncture was received in the thumb of the right hand, from a spicula of bone, during the investigation; but, as this was an accident, the occurrence of which had been several times repeated, without any unpleasant results, in former morbid examinations, the present instance scarcely excited notice. The succeeding day was one of much bodily fatigue, preparing to leave the regiment for a few days, on a hurried visit to London on business of importance; the journey towards which was commenced, by mounting a stage-coach at five o'clock that evening. At midnight, without any premonitory symptom, an acute pain suddenly planted itself in the situation of the puncture in the thumb, which was immediately succeeded by a shivering fit.

The fact of the absorption of virus was now evident, and the anxiety occasioned thereby was excessive. Immediate remedies being requisite, the accidental situation in the midst of a journey became perplexing. Two measures only offered for selection, —either to descend at the first inn upon the road, and immediately to have recourse to remedies among strangers; or to proceed fifty miles further, where, fortunately, the residence of relations and friends offered greater facilities, and rendered success much more probable: the latter alternative was, therefore, adopted.

The period required to reach this wished-for asylum was one of extreme mental anxiety, and acute bodily suffering; the thumb and three first fingers momentarily becoming more painful, with the most violent rigors rapidly succeeding each other. The distance at length accomplished, retirement to a warm bed was instantly adopted, and the assistance of medical friends immediately summoned. For some hours after this period, the most deadly coldness pervaded the whole frame, more especially the lower extremities, which were almost void of feeling and nearly deprived of circulation; while the hand was assuming much tumefaction, and the pain was extending up the fore-arm. After the continuation for a considerable time, with much assiduity, of the application of artificial heat to the stomach and to the lower extremities, a most violent stage of reaction set in. The sensations experienced during this paroxysm were of the most insupportable nature, and certainly bore a stronger affinity to an attack of hydrophobia, than to any other disease to which it could be assimilated. The degree of heat upon the surface was such, that one limb could not be borne in contact with another. Pain in the head was so violent, with such a feeling of internal heat, that the brain appeared on fire. Rapidly succeeding spasms of the diaphragm threatened to suspend respiration.

Deglutition of fluids was rendered most difficult, while thirst was excessive. The stomach became extremely irritable. A severe pain seized upon the loins; the affection of the hand and fore-arm became agonizing; while vivid red streaks in the course of the lymphatics indicated the progress of absorption of the poison. The warm bath at this period produced a profuse perspiration, and was attended with very salutary effects; and the abstraction of blood from the arm by leeches sensibly diminished the pain, and restrained considerably the tension; but these measures, aided by the most direct sedatives and cathartics, appeared to exert their influence but a few hours, when every symptom became exasperated: the pain and tension occupied the whole of the fore-arm, and the inflamed line of the lymphatics reached the inside of the elbow-joint. Recourse was again had to leeches, with immersion in warm water of the whole limb. Successively the paroxysm returned, and more or less relief was invariably obtained by the assiduous repetition of the measure, especially by leeching, and the immersion of the limb in very hot water.

Two days after the commencement of the attack, the axillary glands had become enlarged, and were very tender to the touch. A stage of excitement, and consequently exasperation of all the symptoms, regularly set in every twelve hours; at which time a very severe pain occupied the right side of the thorax, and occasionally rendered respiration difficult; but the progress of the absorption was evidently arrested at the bend of the fore-arm, to which the inflamed lymphatics could be distinctly traced, but no further: here equally was the boundary of the pain and inflamed surface, and the tension above the elbow-joint was comparatively trifling. The anguish of the hand and arm continued, with such an extreme and peculiar degree of nervous excitement, that narcotics failed entirely to induce the least repose.

On the fourth day, the stage of excitement was more tardy in its commencement, and more brief in its decline; but no distinct remission was yet enjoyed. The state of the limb was become stationary; but no absolute amendment could be recognized, nor sleep, for more than a few minutes at a time, obtained. After this period, the greatest disposition was evinced in the limb to form depots of matter, which for many days it was apprehended could not be prevented; but the result has hitherto proved otherwise, though at this moment (five weeks from the occurrence of the accident,) there is some reason to fear a formation of pus is taking place at the root of the thumb.*

After the lapse of the first week, amendment, though slow, was progressive: still the constancy and acuteness of the pain continued to banish repose, except for very short periods, and febrile paroxysms were of frequent, but irregular, occurrence, till the twentieth day from the first attack; after which, some natural sleep was enjoyed, the pain in the hand having considerably abated, and the soreness of the axillary gland disappeared. But the hand remained almost void of external sensation, and totally deprived of motion; with the biliary functions suspended, the stomach in the most irritable state, and the bodily powers entirely prostrate. At the present time (five weeks from the occurrence of the accident), though renovation of the animal powers has been effected to a certain degree, yet the functions are much deranged: the hand has but little sensation, and scarcely any motion; the fingers are strongly disposed to contract into the palm, and the hand to approach the fore-arm; pain is never absent, and a considerable degree of œdema distends the hand and fingers.†

Questions, of some importance to decide, here suggest themselves. Is the constitution more susceptible at one period than another to the absorption of morbid poison? or is there a description of virus more active and virulent than others? One, or probably both these facts, will be found of occasional occurrence; as the individual, who in this case so nearly forfeited his life, and has for the present lost the use of his hand, had been for years engaged in morbid examinations, during which wounds and punctures had frequently occurred, without creating the least anxiety or the most trifling inconvenience.

* At the commencement of the attack, scarifications were freely made in the wounded thumb.

† Upon the inflammatory action in the affected limb declining, gentle friction with the saponaceous liniment was commenced, which was succeeded by a compound of a more stimulating nature, and more subsequently by the strongest volatile applications, alternated with mercurial friction and other deobstruents, assisted by diligent, continued friction, and equal pressure.

